# Integrating Artificial Intelligence into Biomedical Science Curricula: Advancing Healthcare Education

**DOI:** 10.3390/clinpract14040112

**Published:** 2024-07-11

**Authors:** Aarti Sharma, Amal Al-Haidose, Maha Al-Asmakh, Atiyeh M. Abdallah

**Affiliations:** 1 College of Health Sciences, QU Health Sector, Qatar University, Doha 2713, Qatar; 2 Department of Biomedical Sciences, College of Health Sciences, QU Health Sector, Qatar University, Doha 2713, Qatar

**Keywords:** healthcare, higher education, biomedical sciences, curricula development

## Abstract

The integration of artificial intelligence (AI) into healthcare practice has improved patient management and care. Many clinical laboratory specialties have already integrated AI in diagnostic specialties such as radiology and pathology, where it can assist in image analysis, diagnosis, and clinical reporting. As AI technologies continue to advance, it is crucial for biomedical science students to receive comprehensive education and training in AI concepts and applications and to understand the ethical consequences for such development. This review focus on the importance of integrating AI into biomedical science curricula and proposes strategies to enhance curricula for different specialties to prepare future healthcare workers. Improving the curriculum can be achieved by introducing specific subjects related to AI such as informatics, data sciences, and digital health. However, there are many challenges to enhancing the curriculum with AI. In this narrative review, we discuss these challenges and suggest mitigation strategies.

## 1. Introduction

Digital technologies have increasingly been incorporated into society, with the use of the internet, mobile apps, and telehealth now being a central and indispensable part of our daily living and utilization of healthcare services [1]. In this new era, many tasks previously handled by humans are now performed by artificial intelligence (AI) [2]. AI can be considered to have started when Samuel Arthur created the first self-learning program, a game capable of repeatedly competing against itself [3]. Later, the “father” of AI, Marvin Minsky, described AI as a machine that can carry out a task that is considered to require special intelligence from humans [4]. AI can use both pre-programmed rule-based algorithms and data-driven models. Initial AI systems used “if-then” symbolic logic well before the advent of expert systems [5], where the latter asked patients a series of questions based on their responses [5].

Current AI combines clinical knowledge from experts with programs that work in a similar manner to our biological nervous system. The input is provided in the form of images or symptoms related to a particular illness, and the trained system matches the patterns to accurately make a diagnosis [6]. Put another way, AI can be considered an intelligent machine agent that reads, recognizes, and analyzes software and algorithms [7]. The incorporation of AI into various healthcare services has been made possible by advances in computer power, faster networks, and more reasonably priced storage. In several medical specialties such as radiology, obstetrics, neurology, cardiology, pathology, pharmacology, and robotic surgery, AI has progressed to the point that it may be used for triage, illness pattern prediction, and image interpretation [8].

AI has been incorporated into numerous fields outside medicine including engineering, customer services, commerce and business, surveillance, and more. Clinical decision support systems, which were first introduced in the mid-20th century, marked the beginning of the integration of AI into healthcare. Biomedical sciences is a multidisciplinary field that includes biology, genetics, and biotechnology, and it generates “big” data requiring AI expertise to explore the data [9].

Self-learning machines provide data-driven suggestions for any test or treatment that the end user, a clinician or laboratory technician, can accept, reject, or modify based on their heuristic and domain knowledge. This final decision-making by laboratory end users is based on their expertise in (or biases against) AI [10]. In short, future healthcare providers require expertise and education in understanding big data, generated algorithms, cloud computing, and machine learning to support the healthcare sector [10].

The biggest gap is the lack of enough research conducted in the field of biomedical sciences on implementing AI. This shortfall of academic and practical research on AI integration requires deliberate addressing by the curriculum designer in the field of biomedical science. As research is burgeoning in the biomedical field, pedagogical strategies require extensive changes to address the gaps related to AI education. The seamless integration of AI in the curriculum requires not only curriculum development but an expert mentorship, resources, and student motivation to accept the change in academics. Addressing these gaps in the future will support the implementation strategies and provide effective AI-integrated biomedical programs. This in turn will prepare students for the future healthcare sector and demands of the new AI industry. This paper focuses on the emerging need to provide knowledge about the application of AI to biomedical science graduates to provide an expert laboratory workforce prepared for a future healthcare system with embedded AI.

## 2. Methods and Search Strategy

References for this narrative review were identified through a comprehensive search of the PubMed database. The search terms used were “Artificial Intelligence”, “Biomedical Science”, “Curricula”, and “Higher Education”. The search covered all publications from the database’s inception to May 2024. Articles specifically related to higher education in healthcare disciplines were selected for review. The final reference list was curated based on the originality and relevance of the articles to the broad scope of this narrative review.

## 3. AI in Healthcare

AI is impacting every aspect of our lives including in our homes, social lives, learning, education, and patient care and healthcare delivery [11]. The constantly evolving healthcare sector is under pressure to perform optimally due to rising patient healthcare needs, declining funding, an insufficiently equipped workforce, and a shift in emphasis toward chronic illness management [12]. This expanding healthcare system requires additional support to meet increasing clinical and administrative duties [13]. In this context, AI has been considered as a solution to bridge gaps in the system by managing part of the workload while ensuring enhanced healthcare outcomes [14]. The healthcare system and the use of AI were pioneered in 1976, when a computer algorithm was utilized to treat abdominal pain [14]. The current applications of AI in healthcare include administrative assistance, electronic records, patient history taking, hospital admissions to expert medical specialties, and applications in cardiology, hematology, radiotherapy, pathology, and numerous other specialties. AI has also supported the automated screening of diabetic retinopathy, breast cancer, arrhythmias, and tuberculosis [15,16,17,18] (Table 1).

Electronic health records (EHRs) and other health information systems have recently and extensively been implemented in the health sector. Although designed to streamline patient care, administrative and technical challenges have led to an increase in the workload for many healthcare staff [12]. AI can reduce this workload by performing administrative tasks or operating the system on its own [19]. AI can also be used to compile patient records and provide physicians with a concise summary of the patient’s health profile, minimizing the need for manual data screening [20]. AI has extensively been applied to the field of imaging for both and screening and prioritizing patient care using various software [21]. AI has supported lung cancer and heart disease screening by automatically scanning X-rays. In histopathology laboratories, AI can also scan many histological slides and support the classification of breast abnormalities [22,23].

AI is therefore already assisting physicians and healthcare providers to make timely decisions with reduced human error [24]. Automated screening supports healthcare workers by reducing screening times and lowering administrative burden. More recently, AI has made significant contributions to studying the epidemiology of COVID-19, disease pattern prediction, and reducing the healthcare burden [25].

Nevertheless, advances in AI technology and enormous data generation are challenging healthcare systems [26]. Effective AI to deal with big data, EHRs, imaging, biometrics, and remote patient care and monitoring requires data analysis with sophisticated algorithms [27]. The support of AI in the healthcare sector requires an understanding of the basic algorithms and analysis of data to reduce workload. Therefore, future health workers must be trained in basic AI concepts to correctly interpret the algorithms run by software.

## 4. Impact of AI on Laboratory Settings during COVID-19

Laboratory technicians were overburdened with the high volume of COVID-19 testing, which delayed routine test results. However, AI changed laboratory workflow during the COVID-19 pandemic. AI acted as a support for understanding the enormous data generated and supported healthcare workers in understanding the pattern of disease [28]. The epidemic accelerated technological advances, especially in AI [29]. Since the pandemic, there has been an increase in the exploration and application of AI and other data analytic tools in a wide range of sectors, including healthcare [30]. As a result, new demands for patient care and the clinical laboratory have demanded that laboratory personnel adapt to AI for a new futuristic laboratory workflow [28].

Even after the pandemic, healthcare has remained overworked, understaffed, and faces numerous obstacles, including the need to reduce expenses and burnout among professionals and prepare for future pandemic threats. AI helped healthcare during the COVID-19 pandemic and continues to do so by decreasing the burden on healthcare personnel, replacing humans in specific jobs, and delivering precise and timely findings [12]. In a few instances, AI has proven essential to healthcare, for example with online appointment scheduling, medication dose calculations, immunization regimens, and alerts about adverse occurrences [31,32,33].

As AI is now an integral part of healthcare, future healthcare workers must gain expertise in various AI applications including machine learning, decision aids, imaging, and laboratory management that support physicians and in turn reduce workload. New examples of routine work automation and the incorporation of AI or machine learning algorithms into new sectors emerge every day [34]. As a constantly evolving field, AI is not limited to the abovementioned areas, and the definition of AI will change as technology advances in the future [35]. In general, AI refers to a machine’s capacity for experience-based learning, adaptability to novel inputs, and human-like task performance [28]. The successful application of AI not only requires an understanding of the technology but also requires confidence in accepting the results of AI. This trust in the results may depend on how confident one is about the datasets or the results of AI. The level familiarity with using AI may have an impact on the perceived credibility of the recommendations, and following the suggestions made by AI requires sufficient trust, which in turn can be built through education [29]. Therefore, a major concern about the successful implementation of AI is the lack of training on the context of data and the input of good data [29]. Therefore, all healthcare professionals require training in AI, which will support the advent of superfast AI techniques and laboratory modernization through the automation of physical sample processing, the generation and reporting of test results, and many other tasks [28]. Furthermore, training laboratory staff in AI can support the analysis of results, suggest the best tests to run on a particular patient, and support doctors in agreeing on the best diagnosis. This will contribute to reducing workload and healthcare efficiency.

## 5. AI in Biomedical Sciences

Advances in AI have the potential to make significant contributions to healthcare, both diagnostic and administrative. AI is playing a particularly important role in diagnostic specialties such as radiology and pathology [36]. In addition, AI is supporting hospital administration and managing patient health records. Due to workforce shortages, AI could support healthcare workers including clinicians, laboratory technicians, and front desk staff.

The concept of a healthcare professional will evolve from being an individual information hoarder to systems-thinking professionals who can acquire, evaluate, and apply information to meet the requirements of a specific person or community [37]. To implement AI in the future, healthcare providers will need to collaborate with data scientists and digital specialists. Therefore, this interprofessional learning must be incorporated into curricula to face the challenge of successfully implementing AI in the future [38].

To train students for the new era, the curriculum must advance and be updated, but the main concern is what knowledge and expertise are needed in AI. Students must understand AI like any other new technology [39], and their ability to integrate and exploit the field in practice would benefit from the application and explanation of the entire AI field. Students should be trained on providing proper inputs, analyzing and interpreting the results, and then explaining the results [39]. Biomedical science students will be employed in the healthcare sector in the near future, and many duties of healthcare workers are now shifting towards management, which is increasing the administrative workload [40]. These increased administrative loads can be reduced by training future healthcare staff on the use of AI applications with clinical documentation, report writing, and laboratory record management [41] (Table 2).

The current National Accrediting Agency for Clinical Laboratory Sciences (NAACLS)-accredited biomedical curriculum offers lab-oriented practical training focusing on clinical aspects of different specialties including pathology, hematology, immunology, urology, and aspects of safety and ethics related to laboratory work. The curriculum currently lacks training on AI [40]. Any expansion of scientific and technological innovations in clinical laboratories results in profound changes in laboratory services, ultimately exerting a significant impact on the expected roles of new graduates [40]. The curriculum traditionally provides competencies in laboratory practice, but the evolving AI field requires the effective use and knowledge of machine learning and data analytics [11,27]. “Big” data from EHRs, imaging, biometrics, multi-omics, and remote monitoring via sensors necessitates the use of sophisticated algorithms. Therefore, the traditional curriculum no longer equips healthcare professionals for the changing needs that demand knowledge management and the effective application of machine learning and data analytics [27]. There is an urgent need to incorporate AI training into the curriculum to provide a skilled and competent laboratory workforce fit for future needs.

Curriculum designers must incorporate training in AI or risk creating an untrained medical healthcare workforce. Future workers are concerned about AI due to the lack of training, which is resulting in the lack of enthusiasm and understanding. According to a study of pathologists, 80% of them agreed that AI should be used in pathology over the next ten years, but, even among the most optimistic cohort, a significant proportion of respondents expressed a variety of worries regarding AI [36]. These concerns and lack of knowledge must be addressed during the training of healthcare workers so that they are ready to adapt AI in the workplace.

There are therefore several academic initiatives in collaboration with engineering colleges to incorporate AI coursework into curricula for future healthcare workers. The University of Toronto has embraced computational coursework for medical students by collaborating with the Vector Institute for Artificial Intelligence and Schwartz Reisman Institute for Technology and Society. The Harvard Medical School has also collaborated with MIT to encourage training for the healthcare workforce on AI. It is important to note that biomedical students who work as teaching assistants (TAs) and manage both administrative and other duties often feel overburdened. Universities are also using AI to ease administrative burdens, with the Georgia University of Technology reporting that many of the queries received by TAs are administrative in nature rather than relating to deeper learning difficulties, so they created an AI program, Jill Watson, to serve as a TA and thereby reduced their burden [42].

## 6. How to Reform Biomedical Education?

The use of AI in healthcare is advancing and has the potential to support healthcare across a range of disciplines. This is particularly evident in diagnostic specialties, pathology and radiology, where AI can assist healthcare workers by providing efficient diagnostic tools for image analysis, diagnosis, and preparing clinical reports [36]. To enhance the predictive and prognostic capabilities of traditional pathology methods, AI-driven diagnostic platforms could conduct image analysis on tissue sections for histopathological examination, assess molecular findings from diagnostic procedures such as next-generation sequencing (NGS), and combine these with clinical and/or radiological data [43]. To incorporate AI in diagnostics, imaging, pathology, and other disciplines, these specialties need competent healthcare workers who have received basic AI training. It is therefore an imperative that policymakers and curriculum designers include AI as supplementary coursework to prepare upcoming graduates in this domain.

The current coursework completed by biomedical students enhances their cognitive, psychomotor, and affective skills [40]; however, understanding computational approaches will help students to become proficient in the application of AI to healthcare [44,45]. The current program requires an extracurricular approach as per the concept of the “reimagined medical school” by Flexner in 1910 [46]. Biomedical science students must have a basic framework of courses and should be supported by specific subjects related to AI [47]. The addition of AI in coursework will enhance the knowledge and basic understanding of the complex AI field. In addition, upgrading the program by adding AI into clinical training will enhance the skills of students and interest in AI-based diagnostics. This expansion of the biomedical science curriculum will help the future healthcare workforce to keep pace with the changing demands of healthcare in the AI era [47].

The biomedical graduate program is constantly reviewed and restructured to achieve workplace-based competency [40]. The current curriculum as per NAACLS requirements is a combination of coursework and clinical training in hospitals [48,49]. The biomedical science curriculum offered to undergraduate students is a well-structured NAACLS-accredited program, but it needs more opportunities for students to keep pace with AI. The curriculum focuses more on skilled laboratory work and clinical training, as preceptors want students to pass the national ASCP certification [50].

**Figure 1:**
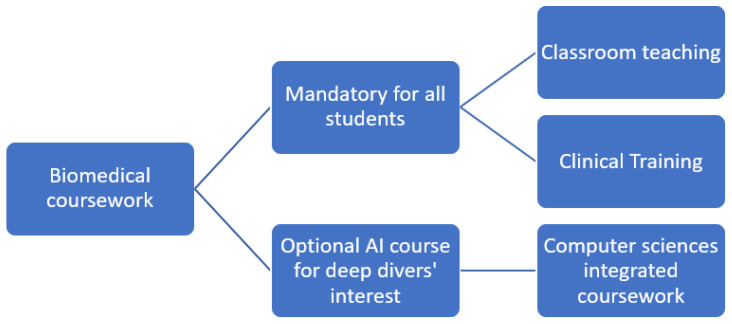
Suggested biomedical science coursework in AI.

Not every student is interested in AI, but interested students should have available additional courses on informatics, digital health, implementation sciences, and data sciences (Figure 1) [51]. These students should have opportunities to work in collaboration with engineering, computing, and interdisciplinary colleges. This interdisciplinary collaboration will enhance competency in clinical decision support systems, EHRs, and advancing AI technologies [10]. In addition, skills on data analytics should be enhanced, as this will increase knowledge and skills on data analysis and data evaluation, which is widely used in AI [10].

Coursework for interested students will require collaboration with other departments including applied computer sciences and computer/software engineering. These additional courses can be delivered during summers or semester breaks. Coursework designers should also support students with targeted funding to establish collaborative research products between biomedical science and computer science departments. This will enhance their skills in practice and prepare future healthcare workers to be more knowledgeable and skilled with AI (Table 3).

## 7. Challenges in Incorporating AI into Biomedical Science Curricula

While AI certainly has promise to support healthcare workers, there are challenges in incorporating AI into health education systems. Recent studies have shown that every field in healthcare is exploring the use of AI for supporting healthcare workers [52], and future developments in AI are anticipated to affect all facets of healthcare [6]. Despite there being many situations in which AI can execute healthcare tasks just as well as or even better than humans, implementation issues will prevent the widespread automation of healthcare professional positions for the foreseeable future [53]. The current lack of training on AI for the healthcare workforce is threatening future advances in AI [14,54]. Due to the global shortage of healthcare professionals, it is challenging for physicians or scientists to devote time and skills to applying AI, further arguing for dedicated and protected training [55].

It is imperative that health science students understand the fundamentals of AI to be able to assess AI-based research and clinical validation and recognize the positive and negative aspects of AI [52]. AI should therefore be included in biomedical schools as a permanent subject in the curriculum. The incorporation of AI education into biomedical coursework might be challenged by the lack of faculty expertise, variable student backgrounds, lack of interprofessional training, and shortages in education resources and funding [54,56]. Faculty members must be knowledgeable about basic AI techniques and applications, so additional interprofessional training and funding might be needed for faculty [52]. As students come from different backgrounds, there is bias in embedding AI into existing coursework, as all students are not IT experts [43]. It will be challenging to incorporate AI into current biomedical curricula, which are already overcrowded with content, without overloading students [57]. Furthermore, ethical issues related to data protection, openness, and responsible AI use need to be taken into consideration [58]. Financial obstacles in purchasing AI software and practical training on this software are additional risks for both management and students [12].

These challenges highlight how difficult it might be to restructure curricula to successfully accommodate AI into the teaching of biomedical sciences, but mitigating these challenges is essential for future healthcare support [59]. Faculty expertise in AI can be improved through multidisciplinary cooperation and interprofessional development programs. Additionally, collaborating with academic institutions, especially in the field of computational sciences, can provide students with access to resources like real-world datasets and computational tools, which can enhance their educational experience [60]. Teaching addressing real-world problems and adding short new AI modules that address ethical issues would encourage responsible AI use and prepare students for ethical dilemmas in professional settings [61]. Lastly, creating mentoring programs based on research with interdisciplinary faculty could help students train in AI and also make it more feasible for AI to be successfully incorporated into biomedical education [62,63]. These approaches could help overcome the challenges associated with integrating AI into biomedical science courses and ensure students have the understanding and competencies needed to effectively address the nexus between AI and healthcare (Table 4).

## 8. Ethics and AI

The ethical and legal dilemmas surrounding AI represent another major concern about its implementation in healthcare. AI can play the role of a physician and would therefore be held to the same ethical standards and expectations as human physicians [61]. Before being used in extensive clinical applications, AI systems need to be certified. There are currently no recognized benchmarks to evaluate the efficacy and safety of AI systems in clinical settings. Furthermore, AI should fulfill the criteria for confidentiality and privacy, which are mandatory in patient care. This implies that AI systems should uphold ethical standards with respect to informed consent, confidentiality, privacy, and decision-making. Most discussions about the ethics of AI center on privacy issues relating to patient data, as the large amounts of data collected and shared for AI make privacy concerns increasingly relevant [64]. As AI requires sharing big data to perform analysis, this raises further concerns about patient privacy [60,65].

As medical regulations are not yet clear on data curation, this poses a major barrier to adopt AI effectively in the health industry [66]. Strategies that have been used to improve privacy preservation include de-identification procedures before releasing clinical data or internal data analysis before sharing the results with external institutions [60]. There are still unanswered questions regarding who is responsible for medical malpractice involving AI applications or medical carelessness caused by the complexity of AI. Current medical rules are not thorough enough to clearly define the lines of accountability in the event of medical errors. When applied to the partial or full use of AI platforms, goods, or services in the provision of healthcare services, this lack of clarity is more serious [66].

According to the “Digital Health Software Precertification (Pre-Cert) Program”, it is the duty of each nation’s legal system to explicitly define who is liable for distortion introduced by AI systems [67]. The patient’s conviction that the physician will always act in their best interest is the most major ethical factor in hands-on healthcare, which requires a doctor–patient connection entwined with many ethical considerations [43]. The ability of AI applications to choose the ethical course of action in each unique situation is limited. The potential lack of empathy from computer-based services is also an important consideration [62]. Another major consideration is whether AI has the capacity to adequately personalize treatment options by taking into account patient feelings and circumstances, as conventional physicians do, to win patient trust and confidence.

## 9. Discussion

AI is the future of healthcare, and this can be achieved with a workforce competent and educated enough to incorporate AI in healthcare. Artificial intelligence holds promise to support each and every field of the healthcare sector including decision-making, big data analysis, and administration [14,56]. Medical schools are accepting the fact that present-day physicians are not knowledgeable in practicing AI as per the standards of care. This necessitates the incorporation of AI in their curriculum to prepare students for meeting future AI-related challenges [54]. It has also been identified that the permeation of AI will affect not only future physicians but every healthcare sector worker [6]. This has altered the plans of nursing curriculum designers, and they are now preparing the future nursing sector to keep pace with future digital technologies [1]. Nursing schools predominately in the USA and now many other regions and countries have already adopted nursing informatics to support future advanced nursing requirements [68,69].

Recent data have shown that all healthcare specialties are exploring the usage of AI [52]. It has been estimated that in the future, AI will permeate in all healthcare sectors completely [6]. This further supports the requirement to incorporate AI in the biomedical curriculum and train biomedical students to keep pace with future healthcare needs. Recent studies conducted among biomedical students provided insight into the lack of dedicated AI coursework in the existing curriculum [70]. In addition, a survey among biomedical students reflected the increased interest among students to learn AI, which requires a more structured approach towards the curriculum [70]. As for universities worldwide, the University of Toronto has embraced computational coursework for medical students by collaborating with Vector Institute for Artificial Intelligence and Schwartz Reisman Institute for Technology and Society. Harvard Medical College has also collaborated with MIT to encourage AI in the healthcare workforce. Curriculum designers must transform their biomedical curricula by collaborating with computer science departments to avoid the risk of creating an untrained medical healthcare workforce.

The incorporation of AI has raised various concerns, among which one of the issues is losing jobs in the future. A survey conducted among biomedical students showed that 40% of students were concerned about losing their jobs in the future [70]. Similar findings were found among pathologists and radiologists in various surveys conducted related to the incorporation of AI in the medical field [71,72]. The lack of confidence in AI with concerns about jobs reflects the lack of knowledge of AI among healthcare sector workers. The complete awareness of the subject will enlighten students that AI aims at re-engineering jobs and not displacing jobs. This interdisciplinary approach while designing curricula will help biomedical students to leverage AI tools effectively in the future. This proactive approach will equip students with the skills and knowledge of AI so that they can responsibly apply their knowledge in the future healthcare sector.

## 10. Limitations

Our narrative review has several limitations. Firstly, as it is authored by four individuals, it is subject to potential bias, including the influence of the authors’ personal viewpoints and their experiences at Qatar University. Additionally, we encountered a scarcity of information specifically related to biomedical science and artificial intelligence compared to more extensively studied fields such as medicine and nursing. However, it is important to note that this is a rapidly evolving research area, and new insights may emerge soon, potentially rendering some of our comments outdated.

## 11. Conclusions

AI has significant potential, and if it is successfully applied in biomedical sciences, it could enhance student expertise. The regulatory bodies of each nation implementing AI need to develop curriculum amendments that can be integrated into current training. Future biomedical students can become more knowledgeable healthcare professionals by being familiar with AI through the incorporation of AI into their coursework. However, many questions arising out of the use of AI must be answered in a transparent manner. This call to action cannot be dismissed through skepticism; in fact, many AI-related problems make the argument for effective student learning significantly stronger. Although AI in healthcare has a lot of potential, successful integration in biomedical curricula is still challenging. Ongoing research and development in AI should focus on creating adaptable and comprehensive educational frameworks. Collaborative efforts between academic institutions, industry leaders, and regulatory bodies are essential to develop standardized guidelines and best practices for AI integration taking into account the rapidly evolving nature of this field. Addressing the challenges summarized in this review, future biomedical students will be well equipped to leverage AI in their professional practice.

## Figures and Tables

**Table 1:** The benefits of the incorporation of AI into healthcare.

Diagnostic accuracy	The incorporation of AI in diagnostics during COVID-19 increased the accuracy of diagnosis through faster and accurate image analysis
Laboratory workflow support	AI reduced the workload for healthcare providers by providing automation support for sample analysis and, later, speedy data analysis
Research and development	The incorporation of AI into healthcare served as the most efficient tool to discover and apply antiviral drugs and vaccines by the interpretation of big patient data
Patient triage optimization	AI supported patient triage by prioritizing critical cases depending on the available data
Quality assurance	The accuracy and reliability of testing were monitored by AI, resulting in reduced system error and delivering high quality work in laboratories

**Table 2:** Potential benefits of empowering biomedical science students with AI.

Radiology imaging and diagnostic approach	AI and machine learning training can support biomedical science students in using algorithms to read images of X-rays and CT and MRI scans to reach an accurate diagnosis
Precision medicine	Biomedical science students can support precision medicine by analyzing big datasets and providing tailored medicinal or genetic approaches to patients
Reducing workload	Efficient AI incorporation in healthcare can help future biomedical science students to reduce their workload as they will be trained in the automation of databases, imaging, etc.
Research and innovation	AI-trained biomedical science students can be an integral part of new innovations related to research or drug discovery
Career opportunities	AI-trained biomedical science students can explore more career and job opportunities in research and administrative healthcare settings
Strategy planning and disease controls	AI-trained healthcare professionals with a biomedical background can detect diseases at their early stages and estimate their potential to spread. This AI approach supports healthcare in planning strategies to control diseases
Ethical considerations	Ethical considerations are the biggest challenge in applying AI to patients and can be resolved by training biomedical students on the ethics related to AI in patient care

**Table 3:** Recommendations for incorporating AI into the biomedical science curriculum.

Curriculum Additions	Recommendations to Achieve AI Expertise
Biomedical engineering and computational data certification	Collaboration with computer/software engineering college and work on certification. Theoretical knowledge can be supplemented with practical experience with AI.
AI learning groups and open journal clubs	Interested students can receive hands-on training and learning from computer or data science students by sharing common learning groups. Students can exchange views and answer each other’s questions.
AI: fundamental concepts	Students should enhance their basic AI skills such as data handling, analysis, data visualization, and understanding.
AI: research opportunities	Interested students should be exposed to private–public AI services and should be involved in AI research projects. These projects would help students to apply new approaches to their work.

**Table 4:** Challenges in incorporating AI into biomedical science curricula and their mitigation.

Field of Challenge	Challenge	Mitigation
Faculty expertise	The lack of faculty expertise represents the biggest challenge to incorporating AI into existing biomedical coursework. Many have only limited knowledge on AI methodologies and their applications. This lack of expertise acts as a barrier to teaching AI to biomedical students	Training the trainers is the solution to this challenge. CPD on AI should be offered through workshops, seminars, and short-term interdisciplinary AI courses to enhance their AI skills
Curriculum restructuring and AI integration	Amending the current curriculum with additional AI coursework is a challenge, as it might be overwhelming for biomedical students and preceptors	The curriculum should be reviewed thoroughly to find potential areas to incorporate AI into existing biomedical coursework. This can be achieved by adding foundational elective AI courses for students, which might require interdisciplinary faculty review
Variable student backgrounds	Biomedical students have different levels of prior knowledge on AI, posing a challenge to offering different levels of AI coursework to students to match their existing knowledge	Introductory AI modules should be provided in the coursework, but for “deep divers” with more extensive knowledge or interest in AI, more extensive AI coursework and research opportunities could be provided
Practical training in AI	Hands-on training for students on AI requires well-equipped labs with specialized equipment and software	AI training requires a new laboratory infrastructure with the latest software and tools for training. This will provide students with hands-on training on algorithms, data analysis, image processing, etc.
Limited resources	Limited financial resources pose a challenge to equip students with training in the latest AI technologies	Allocated funding and budgeting will support in planning new infrastructure and laboratories. In addition, partnership with different institutions will minimize the cost of AI incorporation
Ethical considerations	Ethical considerations: data mining, data privacy, and the responsible application of AI are a challenge	Case studies and real-world examples related to ethics will encourage critical thinking among students in relation to AI
Assessment methods	Effective and standardized assessment methods for assessing students’ knowledge on AI concepts are lacking	Designing exams, projects, presentations, hands-on assignments, and group projects related to the real-world biomedical application of AI will develop understanding and support student assessment
Interprofessional education (IPE)	Interdisciplinary collaboration between computer sciences, engineering departments, and biomedical sciences is challenging, considering the different objectives and priorities of coursework	IPE can be encouraged by structuring interdisciplinary teams with faculties from both computer science and biomedical backgrounds. In addition, faculty should participate in cross-disciplinary seminars to understand and incorporate AI more effectively in coursework. Interdisciplinary student mentors can also promote IPE by assigning joint AI projects to students
